# Development of 3D-printed artificial muscles for meat analogs: structural and functional changes modulated by ultrasound-assisted glycosylation of rice protein with d-xylose^☆^

**DOI:** 10.1016/j.ultsonch.2026.107947

**Published:** 2026-08-01

**Authors:** Jung Soo Kim, Jiyoon Kim, Kwang-Deog Moon

**Affiliations:** a School of Food Science and Biotechnology, Kyungpook National University, 80 Daehak-ro, Daegu 41566, Republic of Korea; b 3D Food Printing Technology Research Institute, FOODPRO CO., Ltd., 14 Bokhyeon-ro, Daegu 41530, Republic of Korea; c Smart Food Manufacturing Research Group, Korea Food Research Institute, 245 Nongsaengmyeong-ro, Wanju-gun 55365, Republic of Korea

**Keywords:** Ultrasonic time, Maillard reaction, Disulfide bonding, Solubility, Structure, Texture

## Abstract

3D food printing enables the fabrication of meat analogs with controlled structures and textures that are difficult to achieve using conventional processing. Therefore, suitable food-inks are essential for 3D-printed meat analogs to ensure precise extrusion, shape retention, and enhanced texture and color development during cooking. Given the inherently low solubility and limited functional properties of rice protein isolate (RPI), chemical and physical modification strategies are required to improve its applicability as a food-ink. In this study, ultrasound-assisted glycosylation was applied to improve the structural and functional properties of protein-based inks for 3D-printed meat analogs. RPI was glycosylated with D-xylose (Xyl) using a horn-type ultrasonic processor operating at 24 kHz and a nominal output power of 500 W under varying ultrasonic treatment times (UT; 0, 5, 30, and 50 min) to produce RPI-Xyl conjugates. Ultrasonic treatment promoted glycosylation and Maillard reaction between RPI and Xyl. Protein-based inks were formulated by adjusting the ratios of soy protein isolate (SPI), RPI, and RPI-Xyl conjugates. Printing parameters were optimized using response surface methodology based on the rheological properties of each ink, after which 3D-printed muscle-like plant protein structures were fabricated as artificial muscles. The structural and functional properties of the artificial muscles varied depending on UT. The Maillard reaction of RPI-Xyl conjugate contributed to meat-like color development in artificial muscles after heat treatment. Ultrasonic treatment altered the secondary and tertiary structures of proteins in glycosylated artificial muscles. Among the treatment conditions, UT30 enhanced protein solubility and disulfide bond formation in the cooked artificial muscles. The textural properties of the 3D-printed artificial muscles were further compared to those of beef steak. Notably, artificial muscles subjected to UT30 exhibited superior water retention, hardness, elasticity, and tensile strength compared to beef steak. In contrast, excessive ultrasonic treatment (UT50) induced partial protein denaturation and aggregation, thereby weakening the functional properties of the artificial muscles. Collectively, these findings demonstrate that ultrasound-assisted glycosylation is a effective strategy for improving the structural and functional performance of 3D-printed artificial muscles, providing approach for developing more realistic and high-quality meat analogs.

## Graphical abstract

**Figure:**
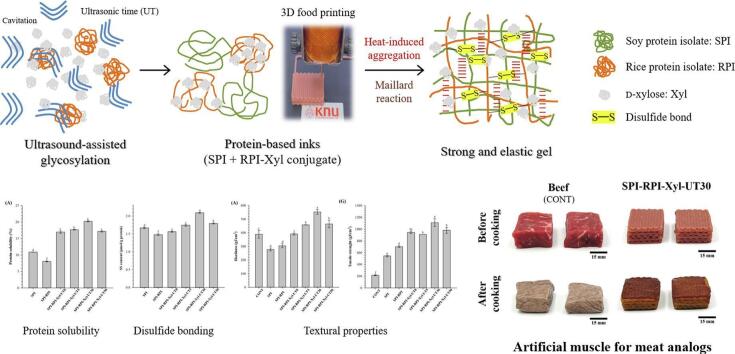

## 1 Introduction

Sustainability, animal welfare, and human health concerns are increasingly shifting consumer attention from conventional meat products toward plant-based meat analogs [1]. Replicating the appearance, texture, and flavor of meat is crucial for improving the quality and consumer acceptance of meat analogs [2]. One of the major challenges in developing plant-based meat analogs is reproducing muscle-like structures, including heterogeneous texture, fibrous organization, and heat-induced quality changes. Therefore, the development of artificial muscles that mimic the texture of meat analogs using plant-based materials and appropriate processing technologies is required. Most previous studies aimed at improving the texture of plant-based meat analogs have primarily focused on extrusion processing [3], [4]. However, extrusion processing, which is typically performed under high temperature and pressure conditions, has limitations in replicating the marbled appearance and diverse textures of beef cuts. Accordingly, many meat analogs remain insufficient in satisfying consumer expectations for meat-like sensory quality.

3D food printing (3DFP) offers a distinct approach because it enables spatial control of food materials through layer-by-layer deposition. Unlike conventional processing, 3DFP can fabricate customized structures by controlling printing patterns, internal architecture, and material distribution [5]. This capability is particularly relevant to meat analogs, in which the target quality involves not only bulk texture but also structural organization contributing to muscle-like appearance and mouthfeel. Therefore, 3DFP can be considered a structure-design strategy for producing meat analogs with controlled geometry and complex texture characteristics. To realize this concept, the development of suitable food-inks is essential. In generally, food-inks should possess rheological properties that allow smooth extrusion through a narrow nozzle while maintaining sufficient self-supporting capacity to preserve 3D shapes structures after printing [6]. For meat analog applications, food-inks should additionally exhibit thermal stability and maintain desirable texture and color changes during 3DFP and subsequent cooking processes.

Soy protein isolate (SPI) is widely used in the production of meat analogs because of its strong gel-forming ability derived from protein aggregation and disulfide bond-mediated cross-linking [7]. Although SPI can enhance the textural properties of meat analogs, its high viscoelasticity restricts the applicable concentration in food-inks, making it challenging to produce meat analogs with the desired texture and printability. Rice protein is a minor component of rice compared with starch, accounting for approximately 6 to 9% of the dry weight [8]. Rice protein isolate (RPI) mainly consists of alkali-soluble glutelin, which represents approximately 80% of total rice protein and contains disulfide-linked structures [9]. However, the main drawback of RPI is its low solubility, which restricts its industrial applicability [10]. Compared to other plant proteins, RPI exhibits a higher least gelation concentration and lower viscoelasticity, limiting its suitability as a food-ink for meat analog applications. Therefore, a formulation strategy that combining the strong gel-forming ability of SPI with structurally modified RPI may improve both printability and the structural quality of 3D-printed meat analogs.

Improvement of the functional properties of plant proteins can be achieved through chemical, physical, and enzymatic modification treatments [11]. Non-enzymatic glycosylation is a reaction in which the carbonyl group of a reducing sugar covalently bonds to free amino groups in proteins. This process modifies the protein’s structure, which also affects its tertiary structure [12]. Protein glycosylation is widely recognized as an initial stage of the Maillard reaction and has been reported to enhance the thermal stability of plant proteins [13], [14]. d-xylose (Xyl) is a naturally occurring low-molecule-weight reducing sugar whose terminal aldehyde group readily reacts with amino groups during the Maillard reaction [15]. However, glycosylation alone may not be insufficient to fully regulate the structural and rheological properties required for printable meat analog systems. Ultrasound is a non-thermal physical processing technology capable of modifying protein structures through acoustic cavitation, shear forces, and localized pressure changes [16]. During ultrasonic processing, cavitation generates intense shear forces and localized pressure, resulting in structural modification of proteins [17], [18], [19]. In addition, reactive free radicals generated by water sonolysis can oxidize free sulfhydryl groups and alter the molecular conformation of globular proteins [20]. Therefore, ultrasound-assisted glycosylation may provide an effective strategy to enhance the reactivity between RPI and Xyl while regulating the structure–function properties of protein-based food-inks. Although ultrasound-assisted glycosylation has been investigated to improve the functionality of plant proteins, its application to 3D-printed meat analogs remains limited. In particular, the relationship among ultrasound-assisted glycosylation, food-ink rheology, printability, and the final quality of structured meat analogs have not yet been fully clarified.

This study aimed to fabricate muscle-like plant protein structures as 3D-printed artificial muscles using SPI-based inks containing RPI-Xyl conjugates prepared through ultrasound-assisted glycosylation. We hypothesized that ultrasound-assisted glycosylation would modify the structural and functional properties of RPI-Xyl conjugates, thereby improving the rheological behavior, printability, and cooked quality of artificial muscles. RPI-Xyl conjugates were prepared under different ultrasonic treatment times and subsequently incorporated with SPI and RPI to formulate protein-based inks with varying compositions for 3D printing. The printing parameters of each protein-based ink were optimized using response surface methodology (RSM) based on rheological properties, and artificial muscles were subsequently fabricated by 3DFP. Finally, the structural, physicochemical, and textural properties of the cooked artificial muscles were evaluated and compared with those of beef steak.

## 2 Materials and methods

### 2.1 Materials

RPI (88.6% protein) and SPI (90.6% protein) were obtained from Listowel (Kerry, Ireland) and Almi GmbH (Vienna, Austria), respectively. Xyl was purchased from ESfood (Gunpo, Korea). Red pigment containing beet red and cacao was acquired from FOODPRO (Daegu, Korea). Beef sirloin steak (CONT; castrated males, 27 months old, 464 ± 20 kg of carcass weight) used to compare the quality characteristics after cooking was purchased from a local market within 7 d post-mortem (Korean meat quality grade 1 + ). All reagents were analytical grade. NaOH and glycerol (Junsei Chemical, Tokyo, Japan), ninhydrin (Wako, Osaka, Japan), Sodium dodecyl sulfate (SDS), 5,5′-dithiobis-2-nitrobenzoic acid (DTNB), bicinchoninic acid (BCA), and bovine serum albumin (Thermo Fisher Scientific, Waltham, MA, USA), 0.5 M Tris-HCl (pH 6.8), tris, and glycine (Biosesang, Seongnam, Korea), O-phthaldehyde (OPA) and EDTA (Sigma-Aldrich, St. Louis, MO, USA), bromophenol blue (Oriental Chemical, Tai Wai, Hong Kong), β-mercaptoethanol (β-ME; Yakuri Pure, Kyoto, Japan), molecular weight marker (Bio-Rad, Hurchules, CA, USA), and HCl and urea (Duksan, Ansan, Korea) were used.

### 2.2 Preparation of glycosylated products treated with ultrasound

The ultrasound-assisted glycosylation reactions were established through preliminary experiments that evaluated various mass ratios of RPI to Xyl (Fig. S1), as well as different ultrasound power levels and times (Figs. S2 and S3). RPI (7%, w/v) was dispersed in distilled water (DW), and the pH was adjusted to 12 using 1 M NaOH. The dispersion was stirred using a shaking incubator (JSSI-300C, JS Research, Gongju, Korea) at 25 °C and 250 rpm for 1 h, followed by readjustment to pH 7 using 1 M HCl. Xyl was then added to the mixture at an RPI:Xyl ratio of 1:2 (w/w) and mixed using a vortex mixer (REAX top, Heidolph Scientific Products GmbH, Schwabach, Germany) at 2,000 rpm for 1 min. Ultrasound-assisted glycosylation was performed using a horn-type ultrasonic processor (BKUP-900 K, Bio Koncision, Gwacheon, Korea) equipped with a 13 mm diameter titanium probe. Ultrasonic treatment was conducted at a fixed frequency of 24 kHz and a nominal output power of 500 W. The reaction mixture (total mass: 26.3 g; 2.1 g RPI, 4.2 g Xyl, and 20 mL DW) was transferred into a 50 mL conical tube with an internal diameter of 27.5 mm prior to ultrasonication. The ultrasonic treatment times were selected based on preliminary experiments evaluating the effects of treatment duration on glycosylation and browning development (Figs. S2 and S3). Sonication was performed in pulse mode (1 min on and 1 min off) to reduce thermal accumulation during treatment, and the net ultrasonic treatment times were set to 0, 5, 30, and 50 min, designated as RPI-Xyl-UT0, RPI-Xyl-UT5, RPI-Xyl-UT30, and RPI-Xyl-UT50, respectively. During ultrasonic treatment, the conical tube was placed in an ice-water bath maintained at 3 ℃, and the sample temperature was maintained below 35 °C to minimize excessive thermal effects associated with prolonged ultrasonication. Following ultrasonic treatment, the mixtures were heated at 95 °C for 1 h to induce the Maillard glycosylation reaction and then immediately cooled in an ice-water bath to prepare the RPI-Xyl conjugates.

#### 2.2.1 Grafting degree (GD) and browning index (BI)

The GD value was determined by OPA method [15] using UV–vis spectrometer (Evolution 201, Thermo Fisher Scientific). 4 mL of OPA reagent was added with 200 µL of RPI-Xyl conjugates, and held for 2 min at 35℃. Absorbance was measured at 340 nm, with values for RPI-Xyl conjugates and RPI solution denoted as A_1_ and A_0_, respectively. The GD was calculated using following formula (Eq. (1)):

(1) $$Graftingdegree\left(\%\right)=\frac{\left(A_{0}-A_{1}\right)}{A_{0}}\times 100$$

The BI values of RPI-Xyl conjugates, diluted to concentration of 1 mg/mL, were measured by absorbance at 420 nm using UV–vis spectrometer (Evolution 201, Thermo Fisher Scientific).

### 2.3 Preparation of protein-based inks

As shown in Table 1, protein-based inks were prepared by combining SPI, RPI, and RPI-Xyl conjugates obtained under different ultrasonic treatment conditions. The maximum SPI concentration that allowed stable 3D printing in the SPI-based ink was 20% (w/v), as determined in preliminary experiments. Red pigment was completely dissolved in DW and then added to SPI, RPI, and/or RPI-Xyl conjugate mixtures. The mixtures were homogenized at 3,600 rpm for 1 min and then transferred into 50 mL Luer-lock syringes for 3D printing.

**Table 1:** Formulations of protein-based inks with varying contents of soy protein isolate (SPI), rice protein isolate (RPI), and d-xylose (Xyl), and different ultrasonic times (UT).

Sample name	Soy protein isolate (SPI, g)	Rice protein isolate (RPI, g)	d-xylose (Xyl, g)	Red pigment (g)	Distilled water (mL)	Ultrasonic treatment
SPI	20	−	−	0.8	100	−
SPI-RPI	20	3.15	−	0.8	100	−
SPI-RPI-Xyl-UT0	20	3.15	6.3	0.8	100	−
SPI-RPI-Xyl-UT5	20	3.15	6.3	0.8	100	500 W, 5 min
SPI-RPI-Xyl-UT30	20	3.15	6.3	0.8	100	500 W, 30 min
SPI-RPI-Xyl-UT50	20	3.15	6.3	0.8	100	500 W, 50 min

### 2.4 Rheological properties

The rheological properties of ink were determined using 20 mm parallel plate of rotational rheometer (HR-10, TA Instruments, New Castle, DE, USA) with gap of 1,000 µm. Viscosity was measured within a shear rate of 0.01 to 100 *sec*^-1^. The obtained data was fitted by Ostwald-de waele power-law equation (Eq. (2)):

(2) $$\eta =K∙\gamma^{\left(n-1\right)}$$

where η is apparent viscosity, K is consistency index, γ is shear rate, and n is flow behavior index.

For the determination of yield stress, amplitude sweep was measured within a shear strain of 0.1 to 100%, at angular frequency of 10 rad/sec and 25℃. The linear viscoelastic region was determined at a strain of 1% by small-amplitude scanning. The frequency sweep was performed within an angular frequency from 0.1 to 100 rad/sec at 25℃, and the storage modulus (G′), loss modulus (G″), and loss tangent (tan δ = G″/G′) were measured.

### 2.5 3D food printing (3DFP) process

3DFP was conducted using a 3D food printer (3DFP 1.0, FOODPRO, Daegu, Korea) that features temperature control from 5‒100℃, a 50 mL Luer-lock syringe, and a stepper motor with 1.2 A and 0.4 Nm torque. Fixed printing parameters were as follows: nozzle tip diameter of 2.3 mm, printing temperature of 25℃, layer height of 1.5 mm, and infill density of 100%. Flow rate and printing speed were optimized according to rheological properties of each ink. Inks in syringe were printed, and 3D models of artificial muscles were generated in G-code.

#### 2.5.1 Response surface methodology (RSM)

RSM was used to generate experimental designs, statistical analysis, and regression model with Minitab statistical software (Version 16, LLC, PA, USA). A face-centered central composite design (FCCD), which models the response with a second-order polynomial, was selected as the fitting model. The experimental design is presented in Table 2 and S1. Independent variables were flow rate (A) and printing speed (B), and response variable was set to 2.5 mm (±1%) as printed linewidth (Y). Each factor had 3 levels, and 13 combinations were generated including cube points (run order, 1–4), axial points (run order, 5–8), and center points (run order, 9–13). Printed linewidth after one-layer printing was measured using digital caliper. The predicted responses were calculated using second-order polynomial equation, and as follows (Eq. (3)):

(3) Y = β_0_ + β_1_A + β_2_B + β_11_A^2^ + β_22_B^2^ + β_12_AB

where Y is response variable for independent variables (A and B); and β_0_, β_1_, β_2_, β_11_, β_22_, and β_12_ are constant coefficients of intercept, linear, quadratic, and interaction terms, respectively. After evaluating model fitness using coefficient of determination (R^2^) and β coefficients, optimized printing parameters were obtained.

**Table 2:** List of variables defined in face-centered central composite design (FCCD).

Variables	Numeric factors	Name (unit)	Minimum	Maximum
Independent	A	Flow rate (%)	25	35
Independent	B	Printing speed (mm/sec)	5	15
Response	Y	Printed linewidth (mm)	2.475	2.525

Minimum and maximum values of A and B for targeted Y differ by formulation of food-inks. Y value is 2.5 mm (±1%).

#### 2.5.2 Artificial muscles structuring

G-coded 3D model of artificial muscle was generated with a rectangular shape (length: 40 mm; width: 30 mm; height: 15 mm) with a 1 × 1 infill grid pattern. Printing precision was evaluated as difference between the size of printed artificial muscles measured using a digital caliper and the designed size.

### 2.6 Cooking conditions and characteristics

Artificial muscles were cooked at 95℃ for 1 h using water bath method. CONT was cooked at 80℃ for 8 min to reach an internal temperature of 75℃ [21]. Afterward, to prevent temperature rise, artificial muscles and CONT were immediately cooled to 25℃ in water bath at 10℃ for 10 min and 3 min, respectively.

### 2.7 Visual appearance and color changes

Visual appearance of CONT and artificial muscles was captured in studio photo box (Shenzhen Puluz Technology, Guangdong, China) equipped with 1,690 lm of LED lighting from two directions. Color values (CIE LAB) were measured using colorimeter (CR-400, Minolta, Osaka, Japan) calibrated with standard white plate (L*=41.79, a*=7.26, and b*=19.34). L*, a*, and b* values indicate lightness, redness, and yellowness, respectively.

Maillard reaction products (MRPs) were prepared according to a modified method from Jung et al. [22]. Briefly, 2 g of sample was homogenized at 10,000 rpm for 1 min with 20 mL of DW. For MRP extraction, the mixture was ultrasonicated using the same horn-type ultrasonic processor at 24 kHz and 720 W for 5 min, then centrifuged at 2,000 × g and 20 °C for 10 min. The absorbance of the diluted supernatant was measured at 290 nm and 420 nm using a UV–vis spectrometer (Evolution 201, Thermo Fisher Scientific).

### 2.8 Structural characteristics

#### 2.8.1 Crude protein and amino acid composition

Crude protein was determined by micro-Kjeldahl analysis. The amino acid composition analysis was conducted using an amino acid analyzer (LA8080, Hitachi, Tokyo, Japan). Lyophilized artificial muscle (0.1 g) was hydrolyzed by adding 6 N HCl (1 mL) and nitrogen charging for 1 min, followed by hydrolysis at 110 °C for 24 h. After hydrolysis, cap was opened and dried at 80 °C for 24 h. The dried sample was dissolved in 0.02 N HCl (2 mL), and then centrifuged at 10,000 rpm for 10 min. The supernatant was filtered by a 0.2 μm membrane filter, and used for analysis. A 20 μL sample filtrate was injected into amino acid analyzer, passed through an ion-exchange resin column (2622 SCPF, 4.6 × 60 mm) to separate various amino acids. The amino acids separated from column at high temperature reacted with ninhydrin to form colored compounds. Among the 18 amino acids, proline was measured for absorbance at 440 nm wavelength, while remaining 17 amino acids were measured at 570 nm wavelength for absorbance to analyze each amino acid.

#### 2.8.2 Sodium dodecyl sulfate–polyacrylamide gel electrophoresis (SDS-PAGE)

Molecular structures of artificial muscle were analyzed using SDS-PAGE, according to modified method from Jiang et al. [23]. The electrophoresis gel was made up of 5% stacking gel and 15% separating gel, respectively. The solution of lyophilized artificial muscle (15 mg/mL) was mixed with 5X sample buffer (40% glycerol, 10% SDS, 2% (w/v) bromophenol blue, and 0.5 M Tris at pH 6.8, and 20% β-ME) at a ratio of 1:4 (v/v), and then heated at 100℃ for 5 min. Subsequently, 12 μL of sample was loaded onto polyacrylamide gels and 5 μL of molecular weight marker (15‒245 kDa) was used for size estimation. Running voltages were set to 150 V and 70 mA. Gel was dyed and decolorized.

#### 2.8.3 Circular dichroism (CD) spectrum measurement

The secondary structure distribution of lyophilized artificial muscles was determined according to method with modification [24]. Briefly, solution of lyophilized sample (1 mg/mL in 5 mM sodium phosphate buffer at pH 7.0) was centrifuged at 10,000 × g and 4℃ for 5 min, and then supernatant was separated. CD spectra were then scanned using CD spectrometer (J-1500, JASCO, Tokyo, Japan) in wavelength range of 200‒250 nm at scan rate of 100 nm/min. Secondary structure content was calculated at the BeStSel webserver (https://bestsel.elte.hu/index.php).

#### 2.8.4 Fourier transform-infrared (FT–IR) spectroscopy

FT**–**IR spectroscopy was performed using FT**–**IR spectrometer (Perkin Elmer, Waltham, MA, USA) equipped with Quest attenuated total reflectance. The spectra were recorded by scanning lyophilized artificial muscle at wavelengths of 4,000 cm^−1^ to 400 cm^−1^, a scan speed of 0.2 cm/sec, and a resolution of 4 cm^−1^.

#### 2.8.5 Scanning electron microscopy (SEM)

The microstructure of artificial muscle was analyzed using scanning electron microscope (SU8220, Hitachi). Lyophilized sample was attached to SEM sample holders and sprayed with platinum at 20 mA for 160 sec under vacuum using an ion sputter coater (E-1030, Hitachi). Images were taken at 500 × magnification.

#### 2.8.6 Protein solubility

Lyophilized artificial muscle (1 mg/mL) was added with DW, then centrifugation was performed at 1,500 × g and 4℃ for 10 min to obtain the supernatant. The protein content in the supernatant was determined by using a BCA assay, and bovine serum albumin was used to establish the standard curve. 125 μL of sample was added to 1 mL BCA reagent, followed by incubating at 37℃ for 30 min. Absorbance at 562 nm was measured using a UV–vis spectrometer (Evolution 201, Thermo Fisher Scientific). Bovine serum albumin was used as a standard curve. Protein solubility was calculated using following equation (Eq. (4)):

(4) $$Proteinsolubility\left(\%\right)=\frac{C_{\sup}}{C_{\mathrm{total}}\times Crudeproteincontent}\times 100$$

where C_sup_ is protein concentration in supernatant, C_total_ is protein concentration in solution.

#### 2.8.7 Sulfhydryl group (–SH) and disulfide bond (S–S)

SH and SS contents were measured using Ellman’s assay with some modifications [25]. 20 mg of lyophilized artificial muscle was mixed with 10 mL Tris-Gly buffer (86 mM Tris, 90 mM glycine, 4 mM EDTA, pH 8). Mixture was incubated at 25℃ for 1 h, followed by centrifugation at 1500 × g for 10 min. 5 mL of obtained supernatant was thoroughly mixed with 50 μL of 4 mg/mL Ellman’s reagent (DTNB dissolved in Tris-Gly buffer) and then incubated for 15 min at 25℃. Absorbance of sample was recorded at 412 nm using a UV–vis spectrometer (Evolution 201, Thermo Fisher Scientific). Total SH content was determined using the same protocol, with the exception that the buffer contained 8 M urea and 5% SDS. SH and SS contents calculated using following equation (Eq. (5), (6)):

(5) $$SHcontent\left(\mu mol/gprotein\right)=\frac{73.53\times A_{412}\times D}{C\times Crudeproteincontent}$$

(6) $$SScontent\left(\mu mol/gprotein\right)=\frac{\mathrm{TotalSScontent}-freeSHgroupcontent}{2}$$

where A_412_ is absorbance at 412 nm, D is dilution factor, and C is concentration of sample (mg/mL).

### 2.9 Cooking loss, water holding capacity (WHC), and shrinkage

Cooking loss was taken to be percentage of fluids lost after cooking sample, which was calculated using following formula (Eq. (7)):

(7) $$\mathrm{Cookingloss}\left(\%\right)=\frac{\mathrm{Initialwieght}-Weigthaftercooking}{\mathrm{Initialweight}}\times 100$$

WHC was analyzed based on filter-paper press method [26]. The cooked sample was weighed and placed between two sheets of filter-paper (Whatman #2). After applying a load of 2 kg dumbbell weight, moisture on surface of samples was wiped and weighted, which was calculated using following formula (Eq. (8)):

(8) $$\mathrm{Waterholdingcapacity}\left(\%\right)=\frac{\mathrm{Weightafterpressing}}{\mathrm{Initialweight}}\times 100$$

Shrinkage of samples after cooking was measured with a digital caliper. The length, width, and height shrinkage ratios were calculated as a percentage.

### 2.10 Textural properties

Texture profile analysis (TPA), shear force, and tensile strength of cooked CONT and artificial muscles were conducted with texture analyzer (Compac-Ⅱ, Sunscientific, Tokyo, Japan). TPA recorded hardness, gumminess, springiness, cohesiveness, and brittleness by two-bite compression test using cylindrical probe of 50 mm. Testing conditions were set with pre-test speed of 2 mm/sec, test and post-test speed of 1 mm/sec, compression distance of 3 mm, clearance of 7 mm, and loadcell of 10 Kgf. Shear force was performed using a blade of vee-shaped (60° angle) cutting edge. Testing conditions were set with test speed of 4 mm/sec, compression distance of 15 mm, and loadcell of 10 Kgf. Tensile strength was measured using samples with size of 30 × 40 × 9 cm. Sample was fixed by clamping and stretched 5 mm at test speed of 1 mm/sec.

### 2.11 Statistical analysis

Statistical analysis was performed using Minitab statistical software (Version 16, LLC, PA, USA) and SPSS software (Version 26, SPSS, IL, USA). The experiments were replicated at least three times, and all data are presented as mean ± standard deviation. Student’s *t*-test was used to assess differences between two independent variables, and one-way analysis of variance (ANOVA) and/or Duncan’s multiple range test was used to evaluate differences between more than two variables. Differences of *p* < 0.05 were considered statistically significant.

## 3 Results and discussion

### 3.1 Grafting degree (GD) and browning index (BI)

**Fig. 1:**
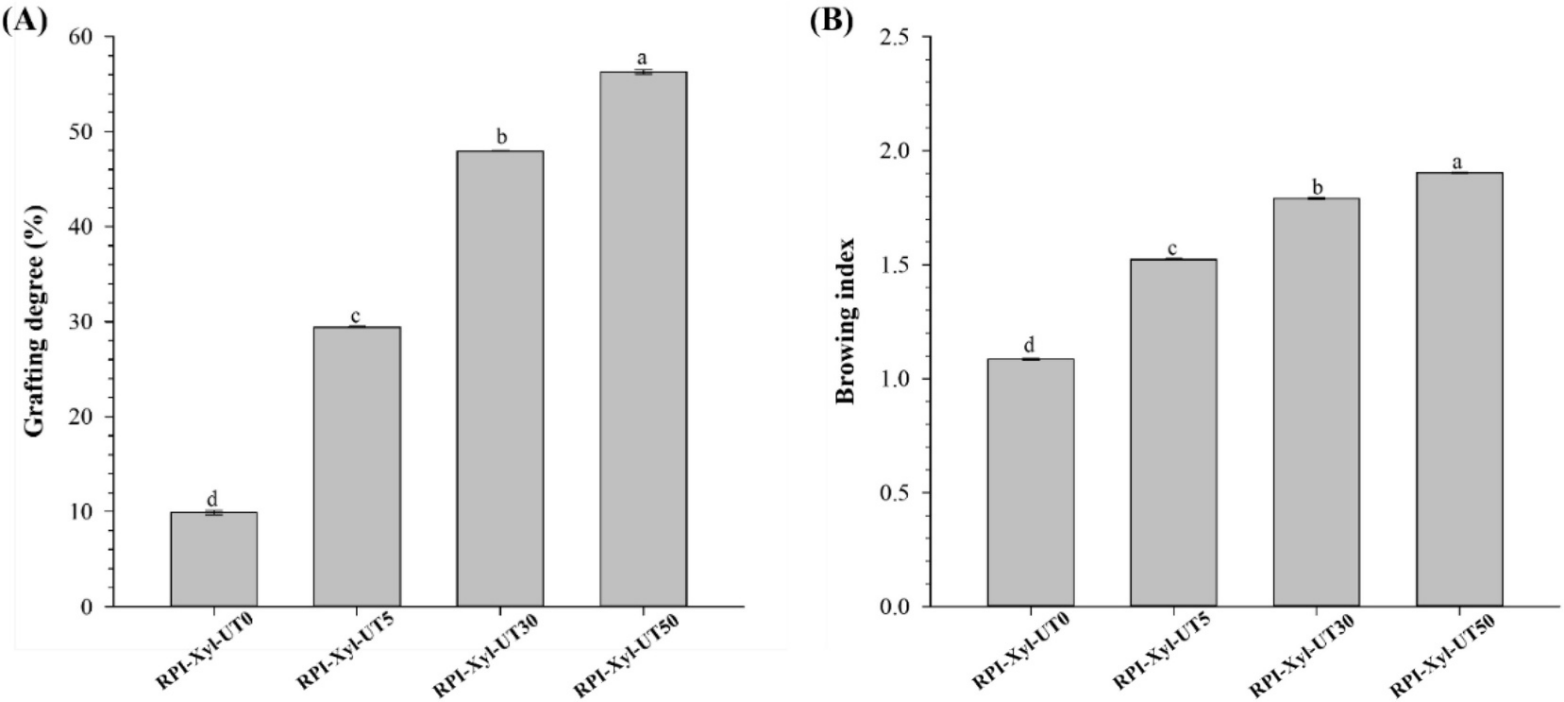
Grafting degree (A) and browning index (B) of glycosylated products of rice protein isolate (RPI) with d-xylose (Xyl) at different ultrasonic times (UT). Values are mean ± SD (n = 3). Different letters indicate significant differences (*p* < 0.05).

The effects of ultrasonic treatments at different durations (0, 5, 30, and 50 min) on the glycosylation of RPI and Xyl were evaluated using grafting degree (GD) and browning index (BI), as illustrated in Fig. 1. The OPA method is widely used to determine the GD between plant proteins and reducing sugars [27], confirming the formation of RPI-Xyl conjugates in all samples (Fig. 1A). The GD values of the conjugates significantly depended on UT, with ultrasound-assisted conjugates exhibiting values more than 2.97-fold higher than those of untreated conjugates. Ultrasound-induced cavitation and shear stress can partially unfold the globular structure of RPI, thereby increasing surface activity and exposing reactive amino groups [28]. Therefore, ultrasonic treatment likely promoted glycosylation with Xyl by increasing the number of accessible reaction sites on RPI. Melanoidins, which are final products of the Maillard reaction, exhibit characteristic absorbance at 420 nm and are commonly used as indicators of glycosylation progression [29]. The BI value of RPI-Xyl conjugates significantly increased after ultrasonic treatment (Fig. 1B), indicating that UT accelerated the formation of the Maillard reaction products. Han et al. [30] also reported that the browning intensity of protein-saccharide conjugates increased with UT, while GD declined after UT 45 min of treatment. Excessive ultrasonic treatment can further unfold proteins, leading to aggregation and a consequent reduction in the number of reactive amino groups available for glycosylation [31]. These results suggest that appropriate UT treatment effectively accelerates glycosylation process and promotes the formation of RPI-Xyl conjugates. Understanding the effects of ultrasonic treatment on structural and functional properties of proteins in glycosylated artificial muscles is essential to assessing the suitability of RPI-Xyl conjugates as materials for meat analog applications.

### 3.2 Rheological properties of protein-based inks

**Fig. 2:**
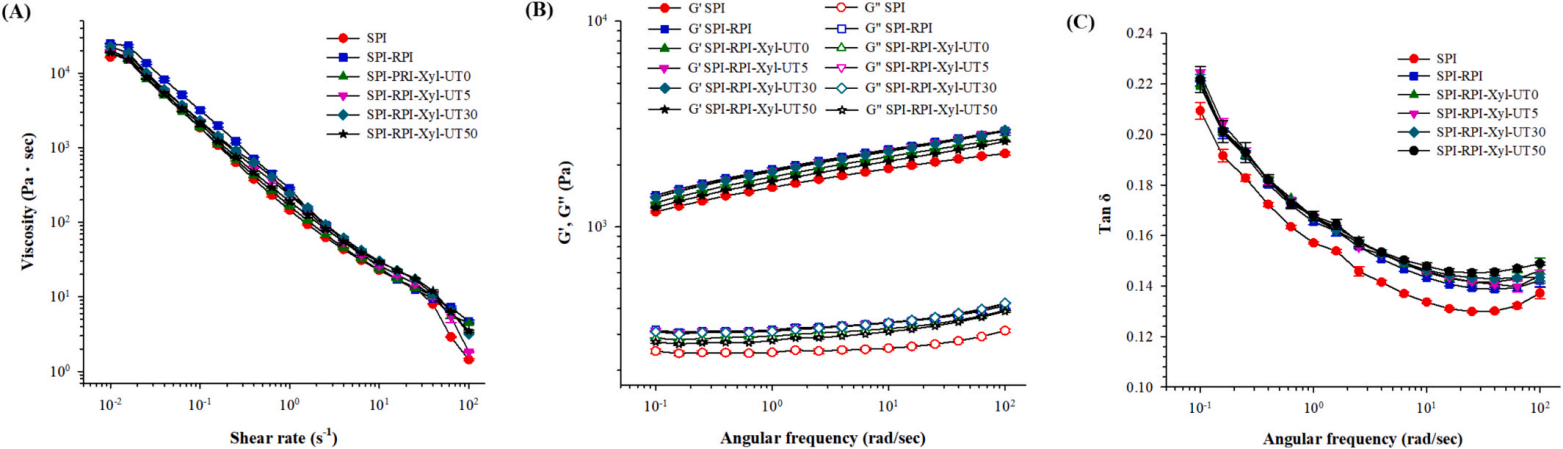
Viscosity (A), storage and loss modulus (B), and loss tangent (C) of protein-based inks with varying contents of soy protein isolate (SPI), rice protein isolate (RPI), and d-xylose (Xyl), and different ultrasonic times (UT). Values are mean ± SD (n = 3).

Extrusion, recovery, and self-support are critical stages in extrusion-based 3DFP; therefore, food-inks must possess suitable rheological properties throughout at all stages. The rheological properties of protein-based inks formulated with different contents of SPI, RPI, and ultrasound-assisted RPI-Xyl conjugates are presented in Fig. 2 and Table 3. For smooth extrusion and structural recovery after deposition, food-inks should exhibit high pseudo-plasticity and shear-thinning behavior [32]. All inks exhibited a continuous decrease in viscosity with increasing shear rate (Fig. 2A), confirming typical shear-thinning behavior. During the self-support stage, the mechanical strength of food-inks plays a critical role in maintaining the stability of the designed 3D structure and and printing pattern. G′ and G″ indicate the elastic and viscous behaviors of food-inks, whereas tan δ (G″/ G′) values below 1.0 indicate elasticity-dominated gel systems [33]. The mechanical spectra of the protein-based inks are presented as double-logarithmic plots (Fig. 2B and 2C). For all inks, G′ values were significantly higher than G″ values, resulting in tan δ values ranging from 0.1372 to 0.2241 across the tested frequency range. These results indicate that all protein-based inks possessed sufficient viscoelastic stability to support smooth extrusion and layer-by-layer deposition during 3DFP.

**Table 3:** Shear-thinning coefficients and yield stress of protein-based inks with varying contents of soy protein isolate (SPI), rice protein isolate (RPI), and d-xylose (Xyl), and different ultrasonic times (UT).

Samples	R^2^	Flow index, n	Consistency index,K (Pa·s^n^)	Yield stress (Pa)
SPI	0.9895 ± 0.0026^a^	−0.97 ± 0.01^b^	203.25 ± 7.56^d^	519.53 ± 9.36^f^
SPI-RPI	0.9904 ± 0.0058^a^	−0.96 ± 0.02^ab^	292.32 ± 5.60^a^	734.69 ± 3.97^a^
SPI-RPI-Xyl-UT0	0.9952 ± 0.0008^a^	−0.95 ± 0.01^ab^	244.42 ± 1.57^c^	669.26 ± 7.93^d^
SPI-RPI-Xyl-UT5	0.9954 ± 0.0012^a^	−0.97 ± 0.01^b^	245.08 ± 3.72^c^	700.63 ± 0.85^c^
SPI-RPI-Xyl-UT30	0.9913 ± 0.0011^a^	−0.96 ± 0.01^b^	259.26 ± 9.26^b^	718.47 ± 5.75^b^
SPI-RPI-Xyl-UT50	0.9932 ± 0.0002^a^	−0.93 ± 0.02^a^	235.25 ± 1.99^c^	650.70 ± 13.07^e^

Values are mean ± SD (n = 3). Values in each column with different letters are significantly different at *p* < 0.05.

The Ostwald-de Waele power law model was sued to quantitatively evaluate the extrudability and stacking stability of protein-based inks (Table 3). The R^2^ values for all inks exceeded 0.98, indicating that the model adequately described the flow behavior of the inks. The extrudability of food-inks is closely related to the values of n, K and yield stress, with yield stress playing a crucial role in supporting deposited layers during printing [34]. Negative n values indicate pronounced shear-thinning behavior, whereas the ink containing RPI-Xyl-UT 50 exhibited a significantly higher n values than the other formulations. The K and yield stress values showed similar trends following the addition of RPI and ultrasound-assisted RPI-Xyl conjugates. The highest and lowest values were observed for the SPI ink and SPI-RPI ink, respectively (*p* < 0.05). The viscosity and mechanical strength of inks generally depend on protein concentration and intermolecular interactions [35]. In contrast, incorporation of RPI-Xyl conjugates reduced the K value compared to SPI-RPI ink, indicating that glycosylation reduced ink’s viscoelasticity. Wang et al. [36] reported that glycosylation significantly decreases protein particle size, thereby influencing viscoelasticity of protein-based inks. The K and yield stress values of inks tended to increase with UT; however, values decreased at UT 50 min. Moderate UT can promote the formation of a more structured and stable fluid system, whereas excessive UT may disrupt the internal structural stability of proteins [37]. These results demonstrate that ultrasound-assisted glycosylation effectively modulates the rheological behavior of high-protein inks. Understanding these rheological differences among protein-based inks is essential for optimizing 3DFP parameters and improving the printing stability of artificial muscles.

### 3.3 Optimization of printing parameters and printing accuracy

**Fig. 3:**
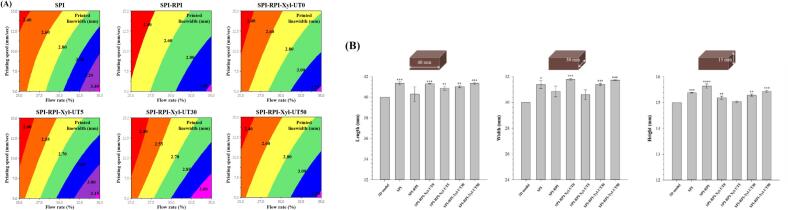
2D contour plots (A) and printing precision (B) of protein-based inks with varying contents of soy protein isolate (SPI), rice protein isolate (RPI), and d-xylose (Xyl), and different ultrasonic times (UT). Values are mean ± SD (n = 3). Asterisks indicate significant differences compared with 3D model dimensions (*p < 0.05, **p < 0.01, ***p < 0.001, ****p < 0.0001).

3DFP parameters for food-inks must be precisely optimized to accurately reproduce the designed printing patterns for meat analogs. RSM is an efficient statistical approach for simultaneously optimizing multiple processing parameters [38]. Consistent printed linewidth is essential for accurate evaluation of printing quality; therefore, flow rate and printing speed are selected as critical parameters in RSM analysis [5]. As illustrated in Fig. 3A, printed linewidths exhibited a positive correlation with flow rate and a negatively correlation with printing speed. Inks with higher K and yield stress values (Table 3) produced narrower linewidths under identical printing conditions. Kim et al. [39] reported that differences in discharge behavior are closely associated with rheological properties related to the extrudability of food-inks. The 2D contour plots visually illustrate the regression equations and clarify the relationships between the response variables and printing parameters, as well as the influence of rheological differences among inks. Table 4 summarizes the adequacy of the models, goodness-of-fit values, and ANOVA results. All second-order polynomial models exhibited *p*-values below 0.0001 and R^2^ values exceeding 0.99, indicating excellent predictive performance for optimizing 3DFP parameters [40]. The *p*-values in the second-order polynomial models confirmed that flow rate had a greater influence on printed linewidth than printing speed for all ink formulations. The printing speed was fixed at 10 mm/sec for all inks to standardized total printing time and minimize quality variation among samples after 3DFP [39]. Accordingly, the flow rate of each ink was optimized to achieve a target printed linewidth of 2.5 mm (SPI: 25.88%; SPI-RPI: 27.73%; SPI-RPI-Xyl-UT0: 26.50%; SPI-RPI-Xyl-UT5: 26.70%; SPI-RPI-Xyl-UT30: 27.25%; SPI-RPI-Xyl-UT50: 26.09%). The printed artificial muscles were compared with the dimensions of the designed 3D model to evaluate printing accuracy (Fig. 3B). The overall dimensional deviation of artificial muscles ranged from 0.03 mm to 1.78 mm, indicating high fidelity. Ainis et al. [35] reported that food-inks with higher K values and yield stresses can produce self-supporting and structurally stable 3D-printed constructs because of increased paste strength. All protein-based inks were successfully fabricated into artificial muscles using the optimized printing parameters, demonstrating sufficient extrudability and mechanical stability for 3DFP applications.

**Table 4:** The RSM results and flow rate optimization of protein-based inks with varying contents of soy protein isolate (SPI), rice protein isolate (RPI), and d-xylose (Xyl), and different ultrasonic times (UT).

Samples	Second-order polynomial equations	Regression (*p*-value)	R^2^	Optimal flow rate, A (%)
SPI	Y = 2.77954^****^+0.36889A^****^-0.18889B^****^ +0.03494A^2**^-0.01172B^2^-0.09750AB^****^	0.0000	0.9926	25.88
SPI-RPI	Y = 2.63184^****^+0.26778A^****^-0.13444B^****^ −0.03644A^2****^+0.00023B^2^-0.03583AB^****^	0.0000	0.9927	27.73
SPI-RPI-Xyl-UT0	Y = 2.73690^****^+0.30389A^****^-0.16500B^****^ −0.04580A^2****^+0.01753B^2**^-0.05583AB^****^	0.0000	0.9955	26.50
SPI-RPI-Xyl-UT5	Y = 2.70874^****^+0.29667A^****^-0.15611B^****^ −0.03724A^2**^+0.01443B^2^-0.05333AB^**^	0.0000	0.9952	26.70
SPI-RPI-Xyl-UT30	Y = 2.67092^****^+0.28944A^****^-0.15111B^****^ −0.03822A^2****^+0.03011B^2****^-0.04250AB^****^	0.0000	0.9956	27.25
SPI-RPI-Xyl-UT50	Y = 2.75494^****^+0.30556A^****^-0.16556B^****^ −0.02897A^2****^+0.00103B^2^-0.03583AB^****^	0.0000	0.9956	26.09

Y, printed linewidth (mm); A, flow rate (%); B, printing speed (mm/sec). Optimized flow rate means when Y value is 2.5 mm (±1%) and B value is 10 mm/sec. *p*-values are significantly different at *p* < 0.05. ^****^p < 0.0001; ^**^p < 0.01.

### 3.4 Visual appearance and color changes

**Fig. 4:**
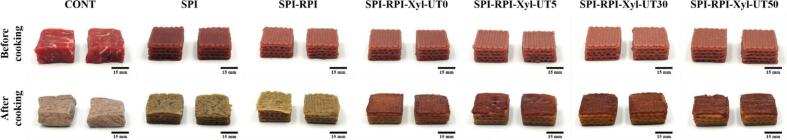
Visual appearance of beef (CONT), and artificial muscles with varying contents of soy protein isolate (SPI), rice protein isolate (RPI), and D-xylose (Xyl), and different ultrasonic times (UT).

3D-printed artificial muscles for meat analogs require careful formulation and processing because thermal stability and color development must be controlled during both 3DFP and cooking. The external morphology of CONT, and artificial muscles containing different contents of SPI, RPI, and ultrasound-assisted RPI-Xyl conjugates before and after cooking is illustrated in Fig. 4. All artificial muscles successfully retained their printed patterns and structural integrity after 3DFP. After cooking, CONT showed pronounced shrinkage and deformation, whereas the artificial muscles maintained their printed structures with slight shrinkage. In conventional meat, changes in redness during cooking are mainly associated with myoglobin denaturation, and sous-vide cooking generally produces a thinner brown surface layer than high-temperature cooking methods. Maillard reaction-induced browning in meat typically occurs above 110℃ [41]. In the artificial muscles, distinct color changes before and after cooking were observed, depending on the addition of RPI and RPI-Xyl conjugates.

Color changes after cooking in CONT, and artificial muscles containing different contents of SPI, RPI, and ultrasound-assisted RPI-Xyl conjugates are presented in Table 5. Artificial muscles containing beet powder were designed to mimic heat-induced color changes observed in CONT. Beet pigments readily lose their red color above 50℃ because of betanin degradation [42]. After cooking, SPI and SPI-RPI samples showed a marked decrease in a* values and increases in both L* and b* values. In contrast, samples containing RPI-Xyl conjugates exhibited a pronounced decrease in L* values, which was attributed to Maillard reaction. The Maillard reaction involves condensation of carbonyl and amino compounds, molecular rearrangement, and polymerization, thereby affecting the flavor, color, and nutritional properties of processed foods [43]. As UT increased, the L* and b* values of artificial muscles tended to decrease, consistent with previous finding showing that ultrasonic treatment altered the color parameters of canola protein isolate [44]. As shown in Table 5, intermediate compounds and brown pigments formed through non-enzymatic browning were detected by UV–vis absorbance at 290 nm and 420 nm, respectively [22]. The MRP values of CONT were the highest (*p* < 0.05), possibly due to the presence of tallow or oxidized tallow [45]. The addition of RPI or RPI-Xyl conjugates significantly increased the MRP values of artificial muscles. Moreover, MRP values increased with UT, which is consistent with the UT-induced increase in the BI values of RPI-Xyl conjugates (Fig. 1B). Glycosylation of plant proteins can contribute to meat-like color development and may improve consumer acceptance of meat analogs [46].

**Table 5:** Color values before and after cooking, and Maillard reaction products (MRP) of beef (CONT), and artificial muscles with varying contents of soy protein isolate (SPI), rice protein isolate (RPI), and d-xylose (Xyl), and different ultrasonic times (UT).

Samples	Before cooking			After cooking			MRP	
	L*	a*	b*	L*	a*	b*	290 nm	420 nm
CONT	40.01 ± 0.69^c^	25.63 ± 0.86^a^	12.76 ± 0.95^a^	38.29 ± 0.80^b^	11.04 ± 0.63^c^	12.27 ± 0.22^d^	1.054 ± 0.011^a^	0.128 ± 0.016^a^
SPI	32.73 ± 0.47^e^	13.14 ± 0.69^e^	6.99 ± 0.69^d^	34.86 ± 0.54^c^	3.69 ± 0.56^e^	11.90 ± 0.25^d^	0.691 ± 0.001^f^	0.048 ± 0.001^d^
SPI-RPI	38.44 ± 0.22^d^	17.94 ± 0.23^b^	11.26 ± 0.41^c^	41.23 ± 0.64^a^	5.45 ± 0.30^d^	17.78 ± 0.26^b^	0.723 ± 0.002^e^	0.061 ± 0.001^c^
SPI-RPI-Xyl-UT0	39.85 ± 0.27^c^	16.48 ± 0.04^cd^	11.33 ± 0.10^c^	33.73 ± 0.84^d^	14.88 ± 0.48^b^	20.70 ± 1.24^a^	0.762 ± 0.001^d^	0.064 ± 0.002^c^
SPI-RPI-Xyl-UT5	42.57 ± 0.13^a^	16.21 ± 0.17^d^	12.46 ± 0.11^ab^	30.52 ± 0.58^e^	15.54 ± 0.38^a^	16.40 ± 0.46^c^	0.790 ± 0.003^c^	0.068 ± 0.002^c^
SPI-RPI-Xyl-UT30	41.41 ± 0.17^b^	16.99 ± 0.18^c^	11.86 ± 0.13^bc^	30.47 ± 1.57^e^	14.77 ± 0.30^b^	16.51 ± 1.52^c^	0.815 ± 0.002^b^	0.081 ± 0.001^b^
SPI-RPI-Xyl-UT50	41.54 ± 0.61^b^	16.26 ± 0.24^d^	11.92 ± 0.23^bc^	29.64 ± 0.26^e^	14.36 ± 0.70^b^	15.66 ± 0.89^c^	0.816 ± 0.002^b^	0.084 ± 0.001^b^

Values are mean ± SD (color values; n = 5, MRP; n = 3). Values in each column with different letters are significantly different at *p* < 0.05. L*: lightness; a*: redness (positive value) and greenness (negative value); b*: yellowness (positive value) and blueness (negative value).

### 3.5 Structural characteristics of artificial muscles

#### 3.5.1 Crude protein and amino acid composition

The crude protein content and amino acid composition of artificial muscles containing different contents of SPI, RPI, and ultrasound-assisted RPI-Xyl conjugates are listed in Table 6. The crude protein contents of SPI and SPI-RPI samples were 87.15% and 87.46%, respectively. Samples containing RPI-Xyl conjugates exhibited crude protein contents ranging from 67.50% to 69.15%, with a tendency to increase as UT increased. Flores-Jiménez et al. [44] reported a slight increase in the protein content of canola protein by UT. Combining legume and cereal proteins can improve plant protein quality by complementing their amino acid profiles [47]. The SPI-RPI sample showed a more balanced overall amino acid profile than the SPI sample. At the same protein content, glycosylated samples exhibited significantly higher total amino acid content than the non-glycosylated samples. Arginine (Arg), lysine (Lys), cysteine (Cys), and proline (Pro) are major amino acids involved in the Maillard reaction of RPI [15]. As UT increased, Arg and Pro contents in artificial muscle decreased, whereas Lys and Cys initially decreased and then increased at UT 50 min. Li et al. [48] reported that amino acids involved in glycosylation increased after UT 40 min, although the glycosylation degree also increased up to 40 min. Among the glycosylated artificial muscles, the sulfur-containing amino acid Cys was lowest at UT 30 min. This reduction in Cys may be attributed to the formation of cystine-derived crosslinks [48]. Additionally, glycosylation increased the content of polar amino acids in artificial muscles. Exposed polar amino acids can enhance protein solubility through hydrogen bonding and electrostatic interactions [49]. The UT-induced variation in amino acid composition is expected to alter the secondary and tertiary structures of proteins in artificial muscles [50].

**Table 6:** Crude protein and amino acid composition of artificial muscles with varying contents of soy protein isolate (SPI), rice protein isolate (RPI), and d-xylose (Xyl), and different ultrasonic times (UT).

Samples	SPI	SPI-RPI	SPI-RPI-Xyl-UT0	SPI-RPI-Xyl-UT5	SPI-RPI-Xyl-UT30	SPI-RPI-Xyl-UT50
**Crude protein (%)**	87.15 ± 0.48^a^	87.46 ± 0.50^a^	67.51 ± 0.53^c^	67.50 ± 0.50^c^	68.91 ± 0.57^b^	69.15 ± 0.56^b^
***Essential amino acids (EAA, mg/g)***						
Lysine (Lys)	21.23 ± 0.06^d^	21.51 ± 0.08^c^	25.36 ± 0.12^a^	25.48 ± 0.11^a^	25.06 ± 0.06^b^	25.40 ± 0.19^a^
Leucine (Leu)	45.61 ± 0.15^b^	47.26 ± 0.13^a^	41.05 ± 0.02^e^	41.29 ± 0.08^d^	40.94 ± 0.08^e^	41.83 ± 0.05^c^
Methionine (Met)	25.28 ± 0.31^d^	28.17 ± 0.02^c^	30.15 ± 0.02^a^	29.87 ± 0.04^b^	29.75 ± 0.03^b^	29.88 ± 0.11^b^
Valine (Val)	120.07 ± 0.56^d^	119.86 ± 0.56^d^	151.04 ± 0.03^b^	154.49 ± 0.99^a^	151.66 ± 0.41^b^	148.69 ± 0.04^c^
Isoleucine (Ile)	26.46 ± 0.29^c^	27.00 ± 0.26^c^	33.58 ± 0.40^b^	34.87 ± 0.47^a^	33.07 ± 0.43^b^	33.20 ± 0.75^b^
Threonine (Thr)	14.69 ± 0.24^e^	14.67 ± 0.20^d^	18.30 ± 0.29^ab^	19.20 ± 0.00^a^	18.56 ± 0.29^c^	18.32 ± 0.02^bc^
Phenylalanine (Phe)	37.10 ± 0.48^e^	37.66 ± 0.09^d^	70.45 ± 0.13d^ab^	70.59 ± 0.05^a^	69.71 ± 0.01^c^	70.08 ± 0.10^bc^
Histidine (His)	36.13 ± 0.10^d^	36.15 ± 0.05^d^	41.71 ± 0.02^a^	41.69 ± 0.01^a^	41.34 ± 0.07^c^	41.55 ± 0.08^b^
**Sum of EAA**	**326.56 ± 2.18^e^**	**332.28 ± 0.30^d^**	**411.65 ± 0.41^b^**	**417.47 ± 0.98^a^**	**410.09 ± 0.66^bc^**	**408.95 ± 0.70^c^**
***Non-essential amino acids (NEAA, mg/g)***						
Tyrosine (Tyr)	8.54 ± 0.05^b^	9.39 ± 0.00^a^	7.83 ± 0.12^c^	6.29 ± 0.11^e^	6.10 ± 0.01^f^	6.89 ± 0.13^d^
Cysteine (Cys)	70.63 ± 0.35^d^	72.60 ± 0.04^b^	75.97 ± 0.23^a^	71.03 ± 0.13^c^	67.82 ± 0.02^e^	72.48 ± 0.26^b^
Serine (Ser)	11.81 ± 0.15^c^	11.81 ± 0.00^c^	27.85 ± 0.44^b^	28.81 ± 0.16^a^	28.82 ± 0.02^a^	28.09 ± 0.01^b^
Alanine (Ala)	20.53 ± 0.04^d^	59.81 ± 0.03^c^	63.02 ± 0.13^b^	63.27 ± 0.02^a^	63.00 ± 0.17^b^	62.81 ± 0.21^b^
Glutamic acid (Glu)	58.34 ± 0.38^d^	58.21 ± 0.01^d^	68.09 ± 0.27^b^	68.90 ± 0.07^a^	67.88 ± 0.18^bc^	67.44 ± 0.39^c^
Aspartic acid (Asp)	74.32 ± 0.01^d^	72.10 ± 0.24^e^	80.28 ± 0.11^c^	81.06 ± 0.04^ab^	81.24 ± 0.44^a^	80.48 ± 0.63^bc^
Arginine (Arg)	60.67 ± 0.37^c^	61.33 ± 1.09^c^	98.12 ± 0.60^a^	97.98 ± 0.17^a^	96.57 ± 0.14^b^	96.19 ± 0.16^b^
Proline (Pro)	2.46 ± 0.01^e^	2.75 ± 0.06^e^	66.00 ± 0.40^a^	64.77 ± 0.20^b^	64.00 ± 0.06^c^	62.97 ± 0.23^d^
**Sum of NEAA**	**307.30 ± 0.32^f^**	**348.00 ± 0.81^e^**	**487.16 ± 1.29^a^**	**482.11 ± 0.63^b^**	**475.42 ± 0.67^d^**	**477.34 ± 1.84^c^**
**Total AA**	**633.87 ± 2.50^d^**	**680.27 ± 0.51^c^**	**898.81 ± 1.22^a^**	**899.59 ± 0.65^a^**	**885.51 ± 0.79^b^**	**886.28 ± 1.42^b^**

Values are mean ± SD (n = 3). Values in each column with different letters are significantly different at *p* < 0.05.

#### 3.5.2 Sodium dodecyl sulfate–polyacrylamide gel electrophoresis (SDS-PAGE)

**Fig. 5:**
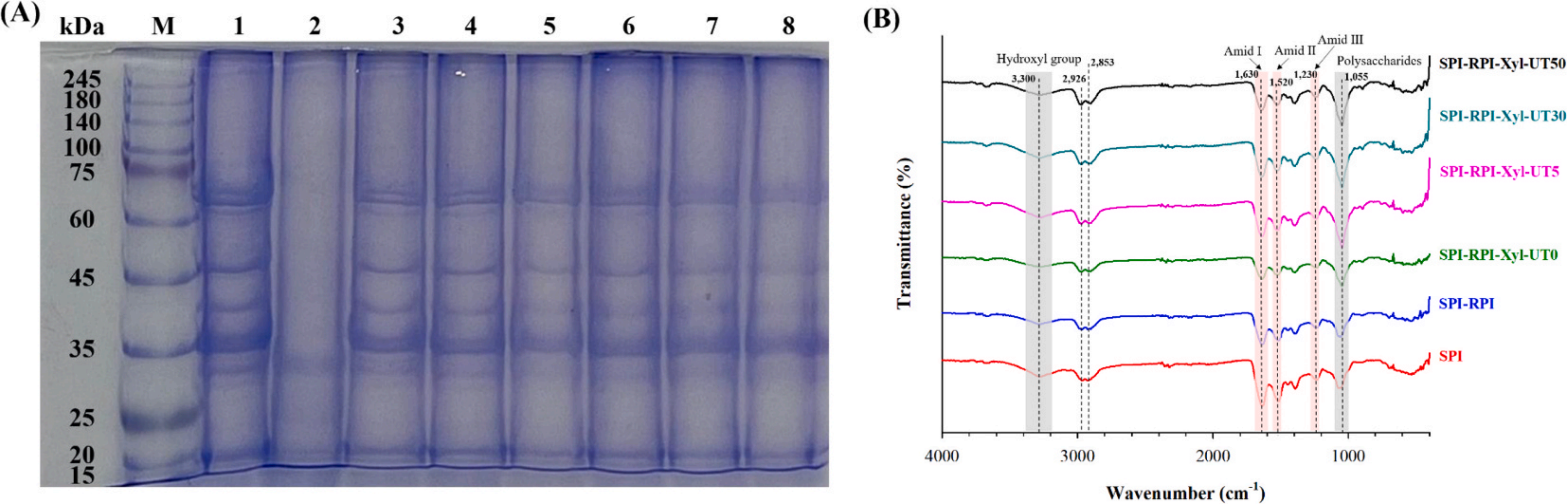
SDS-PAGE profiles (A) and FT–IR spectrum (B) of cooked artificial muscles with varying contents of soy protein isolate (SPI), rice protein isolate (RPI), and D-xylose (Xyl), and different ultrasonic times (UT). M: standard protein marker; line 1: raw SPI; line 2: raw RPI, line 3–8: artificial muscle samples (SPI, SPI-RPI, SPI-RPI-Xyl-UT0, SPI-RPI-Xyl-UT5, SPI-RPI-Xyl-UT30, SPI-RPI-Xyl-UT50).

The SDS-PAGE patterns of cooked artificial muscles containing different contents of SPI, RPI, and ultrasound-assisted RPI-Xyl conjugates, along with raw SPI and raw RPI are presented in Fig. 5A. Raw SPI (line 1) and raw RPI (line 2) displayed typical SDS-PAGE profiles corresponding to their major protein subunits. β-conglycinin and glycinin are the main storage proteins in SPI globulins, accounting for 34% and 41.9% of the total protein content, respectively [51]. β-conglycinin consist of three subunits: α (67 kDa), α′ (71 kDa), and β (50 kDa). Glycinin is a hexamer composed of five subunits, including acidic subunits A (35 kDa) and basic subunits B (20 kDa), which are linked by disulfide bonds [52]. Glutelin, which accounts for 79‒83% of the major protein fraction in RPI, consists of two major polypeptide subunits: 30‒40 kDa (α or acidic) and 19–23 kDa (β or basic). These subunits polymerize via disulfide bonds to form macromolecular complexes [53]. SPI (line 3) and SPI-RPI (line 4) samples showed subunits patterns similar to those of raw SPI and RPI, although the band intensities were weaker. The characteristic protein band pattern can gradually weaken or disappear after heat treatment [14]. Although the electrophoretic patterns of samples containing RPI-Xyl conjugates (lanes 5–8) were generally similar, several protein bands displayed weaker intensities than those of the other artificial muscles. Glycosylation can weaken or eliminate bands corresponding to native proteins, while ultrasonic cavitation can fragment protein molecules through turbulence and high shear forces [43], [54]. A prominent band was observed at the top of resolving gel in samples subjected to ultrasonic treatment, which was consistent with the SDS-PAGE patterns of RPI-Xyl conjugates treated by UT (Fig. S4A). Cheng et al. [55] reported similar results, suggesting that high-molecular-weight compounds can be formed through conjugation between proteins and polysaccharides. These results indicate that ultrasound-assisted glycosylation modified the protein structures of the artificial muscles.

#### 3.5.3 Circular dichroism (CD)

The changes in protein secondary structures of cooked artificial muscles containing different contents of SPI, RPI, and ultrasound-assisted RPI-Xyl conjugates are listed in Table 7. The secondary structure of the SPI sample consisted of 8.45% α-helices, 24.00% β-sheets, 17.00% β-turns, and 50.55% random coils. The addition of raw RPI decreased β-sheets content and increased random coil content of the sample. The incorporation of RPI-Xyl conjugates without ultrasonic treatment decreased α-helices and increased in β-sheets and random coils (*p* < 0.05). The increase in random coils indicates a more flexible protein structure resulting from RPI unfolding during glycosylation, which contributes to changes in protein functionality [15]. In glycosylated samples, α-helices initially decreased but recovered after UT 30 min, whereas β-sheets decreased initially and then increased after UT 50 min. Accordingly, samples containing RPI-Xyl conjugates exhibited a rise in random coil with UT, followed a decline at UT 50 min. Prolonged ultrasonic treatment can promote structural rearrangement and refolding, thereby reducing random coil structures [56]. These findings confirm that ultrasound-assisted glycosylation induced secondary structural modifications in artificial muscles.

**Table 7:** Content of secondary structures in artificial muscles with varying contents of soy protein isolate (SPI), rice protein isolate (RPI), and d-xylose (Xyl), and different ultrasonic times (UT).

Samples	α-helix (%)	β-sheet (%)	β-turn (%)	Random coil (%)
SPI	8.45 ± 0.15^a^	24.00 ± 0.50^a^	17.00 ± 0.90^b^	50.55 ± 0.25^f^
SPI-RPI	9.15 ± 0.45^a^	19.90 ± 1.60^bcd^	18.25 ± 1.45^ab^	52.70 ± 0.60^e^
SPI-RPI-Xyl-UT0	5.40 ± 1.00^bc^	21.45 ± 1.65^ab^	18.65 ± 2.55^ab^	54.50 ± 0.10^d^
SPI-RPI-Xyl-UT5	4.50 ± 0.40^c^	20.70 ± 2.00^bc^	17.65 ± 1.05^ab^	57.15 ± 0.55^b^
SPI-RPI-Xyl-UT30	4.70 ± 0.00^bc^	17.85 ± 1.65^d^	18.85 ± 2.05^ab^	58.60 ± 0.40^a^
SPI-RPI-Xyl-UT50	5.50 ± 0.30^b^	18.05 ± 0.75^cd^	20.35 ± 0.05^a^	56.10 ± 0.50^c^

Values are mean ± SD (n = 3). Values in each column with different letters are significantly different at *p* < 0.05.

#### 3.5.4 Fourier transform-infrared (FT–IR) spectroscopy

FT–IR spectra of cooked artificial muscles containing different contents of SPI, RPI, and ultrasound-assisted RPI-Xyl conjugates are illustrated in Fig. 5B. The broad peak near 3,300 cm^−1^ indicates O‒H stretching vibrations [39]. At 2,926 cm^−1^ (C‒H stretching vibration of CH_3_) and 2,853 cm^−1^ (C‒H stretching vibration of CH_2_) [57], two peaks were presented in all samples. These peaks exhibited changes in intensity after ultrasound-assisted glycosylation, which may be associated with enhanced C‒H stretching vibrations of CH_3_ and CH_2_ groups in polysaccharide chain [58]. Changes in protein structure can be analyzed based on the amide Ⅰ region (1,600‒1,700 cm^−1^, ‒CH_2_ absorption band), amide Ⅱ region (1,450‒1,550 cm^−1^, N‒H bending and C‒N stretching), and amide Ⅲ region (1,200‒1,300 cm^−1^, C‒N and C‒O stretching and N‒H bending) [15]. Samples containing RPI-Xyl conjugates exhibited UT-dependent differences in the peak intensity of amide Ⅰ region. In addition, covalent bonding induced by the Maillard reaction influenced amide group vibrations, resulting in differences in the amide Ⅱ and Ⅲ regions [59]. These spectral changes are closely related to modifications in the secondary structure of proteins [10]. Furthermore, the spectral changes indicate the consumption of C‒O and N‒H groups and the formation of glycosylated products during the Maillard reaction [60]. As shown in Fig. S4B, FT‒IR spectrum of pure Xyl showed characteristic peaks at 3,200 cm^−1^ to 3,500 cm^−1^ and 1,000 to 1,070 cm^−1^, corresponding to hydroxyl groups (O‒H stretching) and C‒O‒C bending, respectively [39]. Artificial muscles underwent a slight blue shift due to ultrasound-assisted glycosylation at 3,300 cm^−1^ peak. At the peak near 1,055 cm^−1^, the addition of RPI-Xyl conjugates caused a clear blue shift and increased peak intensity. These peaks changes indicate that sugar molecules were covalently bonded to RPI, increasing the number of ‒OH groups [15].

#### 3.5.5 Microstructure

**Fig. 6:**
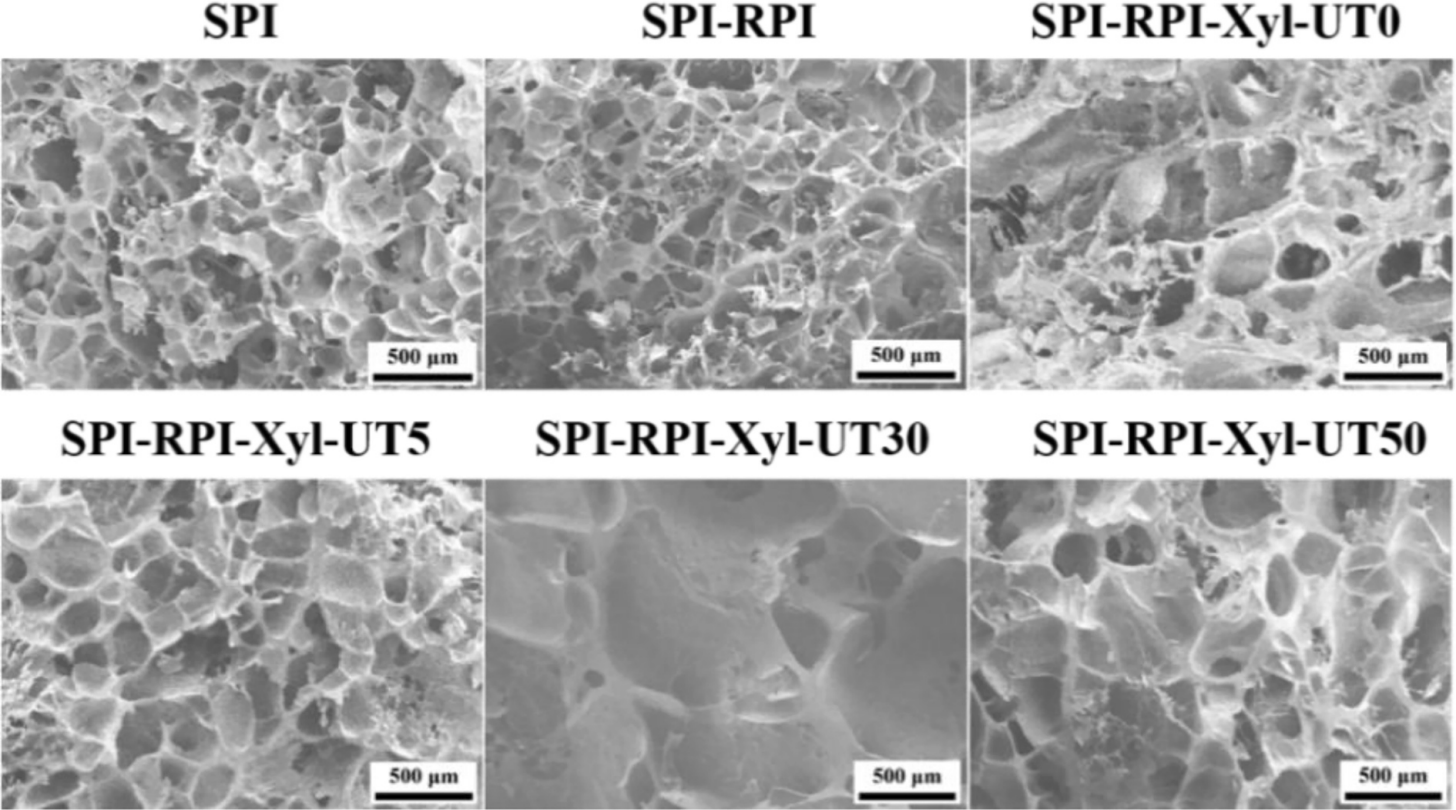
Scanning electron microscopy (500 μm scale bar) of cooked artificial muscles with varying contents of soy protein isolate (SPI), rice protein isolate (RPI), and D-xylose (Xyl), and different ultrasonic times (UT).

The microstructure of cooked artificial muscles containing different contents of SPI, RPI, and ultrasound-assisted RPI-Xyl conjugates observed by SEM is shown in Fig. 6. All samples exhibited porous and irregular network structures. The SPI and SPI-RPI samples showed heterogeneous microstructures, which may be attributed to the denaturation and aggregation of raw SPI and RPI during cooking [9]. Samples containing RPI-Xyl conjugates exhibited smoother and flatter surfaces, despite the presence of pores. After the Maillard reaction, protein structures can unfold, allowing polymer chains to diffuse outward and polysaccharide moieties to aggregate on protein surface [31]. Furthermore, ultrasound-assisted glycosylation promoted the formation of denser network structures in artificial muscles. As the UT increased, the degree of cross-linking in the conjugate gel increased, resulting in a more homogeneous and compact microstructure [59]. The sample treated at UT 30 min showed the most continuous network structure, indicating improved structural cohesion. However, the sample treated with UT 50 min exhibited a coarser gel matrix. These results suggest that excessive ultrasonic treatment can disrupt gel network formation, thereby reducing structural stability [58].

#### 3.5.6 Protein solubility

**Fig. 7:**
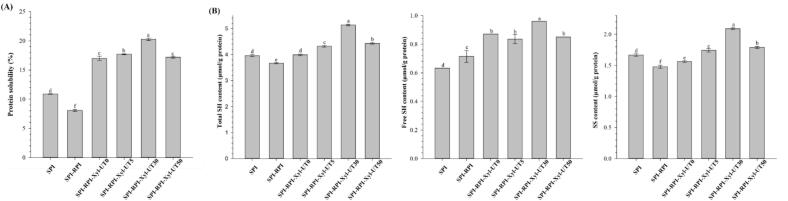
Protein solubility (A) and sulfhydryl group and disulfide bond (B) of cooked artificial muscles with varying contents of soy protein isolate (SPI), rice protein isolate (RPI), and d-xylose (Xyl), and different ultrasonic times (UT). Values are mean ± SD (n = 3). Different letters indicate significant differences (*p* < 0.05).

The protein solubility of cooked artificial muscles containing different contents of SPI, RPI, and ultrasound-assisted RPI-Xyl conjugates is shown in Fig. 7A. Protein solubility is a practical indicator of protein denaturation and aggregation and is widely used to evaluate protein functionality [61]. The solubility of the SPI sample was 10.88%, whereas the addition of raw RPI significantly decreased protein solubility. The low solubility of rice glutelin can be attributed to its structural characteristics, including extensive aggregation and disulfide bond-mediated cross-linking, which reduce protein flexibility and surface activity [62]. The solubility of samples containing RPI-Xyl conjugates significantly enhanced, ranging from 16.99‒20.25%. This increase may be attributed to the higher content of polar amino acids (Table 6) and hydroxyl groups in the organic functional groups (Fig. 5B) induced by glycosylation. Ultrasound-assisted treatment further increased the solubility of glycosylated samples, whereas excessive treatment reduced solubility. Accordingly, the highest solubility value was observed at UT 30 min (20.25%), which decreased to 17.17% at UT 50 min (*p* < 0.05). Moderate ultrasonic treatment can induce protein unfolding and soluble aggregate formation, thereby enhancing protein-water interactions and improving solubility [30]. In contrast, excessive ultrasonic treatment can reduce protein solubility by disrupting covalent bonds through intense cavitation and shear forces [63].

#### 3.5.7 Sulfhydryl group (‒SH) and disulfide bond (S‒S)

The changes in the total ‒SH, free ‒SH, and S‒S content of cooked artificial muscles containing different contents of SPI, RPI, and ultrasound-assisted RPI-Xyl conjugates are presented in Fig. 7B. Free ‒SH groups and S‒S bonds are important functional groups that regulate protein spatial conformation and functional properties through reversible conversion [64]. The total ‒SH and S‒S content in SPI-RPI sample was lower than in SPI sample, while free ‒SH content was higher. The increase in free ‒SH groups may indicate the cleavage of S‒S bonds [65]. These results suggest that the simple incorporation of raw RPI disrupted disulfide bond formation in the SPI gel matrix. Samples containing RPI-Xyl conjugates exhibited significantly higher ‒SH group and S‒S contents than the SPI-RPI sample, and these values varied depending on UT. In globular proteins, ‒SH group are often buried inside the protein molecules and are therefore less accessible. Ultrasonic treatment and the Maillard reaction gradually unfolded protein molecules, exposing buried ‒SH groups to the surface and increasing their detectable content [[15], [66]]. As UT progressed, total and free ‒SH contents in glycosylated samples increased up to UT 30 min and then decreased at UT 50 min. Glycosylated samples treated with UT showed significantly higher S‒S contents than the other samples, whereas excessive ultrasonic treatment reduced S‒S bond formation. Therefore, the sample treated at UT 30 min exhibited the highest ‒SH group and S‒S contents (*p* < 0.05). The ‒SH groups in cysteine and methionine can be oxidized to form S‒S bonds [63]. This interpretation is supported by the significantly lowest cysteine content observed in the UT 30 min sample (Table 6). The formation of disulfide bonds is essential for maintaining the tertiary and quaternary structures of proteins [37].

### 3.6 Cooking loss, water holding capacity (WHC), and shrinkage

**Fig. 8:**
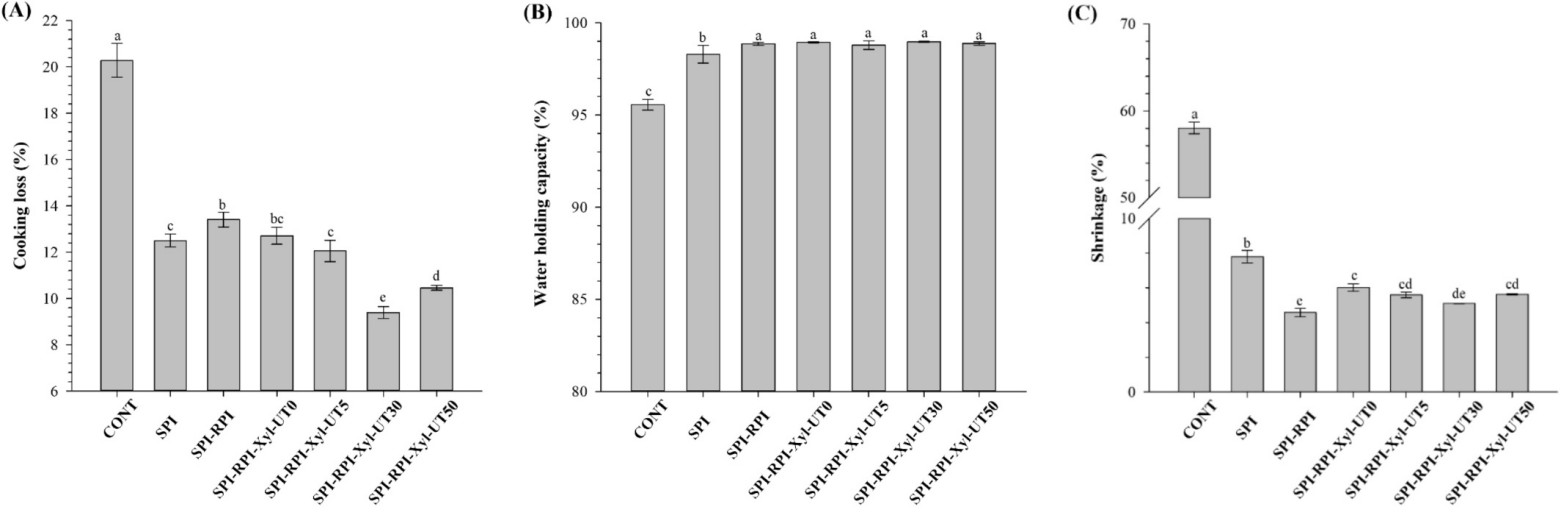
Cooking loss (A), water holding capacity (B), and shrinkage (C) of beef (CONT), and artificial muscles with varying contents of soy protein isolate (SPI), rice protein isolate (RPI), and d-xylose (Xyl), and different ultrasonic times (UT). Values are mean ± SD (n = 3). Different letters indicate significant differences (*p* < 0.05).

Cooking loss, WHC, and shrinkage of CONT, and artificial muscles containing different contents of SPI, RPI, and ultrasound-assisted RPI-Xyl conjugates are shown in Fig. 8. Consumers expect juiciness in beef steak, making water retention an important quality attribute for meat analogs. Cooking loss is normally correlated with juiciness, and juiciness and tenderness are closely associated with each other [41]. The cooking loss of CONT was 20.28%, which was significantly higher than that of artificial muscles, ranging from 9.39% to 13.42% (Fig. 8A). Cross-linking between protein molecules through disulfide bonds during heating can effectively entrap water within the gel matrix [67]. In addition, a uniform and dense microstructure can enhance water retention in protein gels [59]. SPI-RPI-Xyl-UT30 showed the lowest cooking loss (*p* < 0.05), which can be attributed to its more cohesive structure (Fig. 6) and higher S‒S content (Fig. 7B). WHC reflects the ability of a gel system to prevent water loss or drainage from gel network under external pressure [68]. CONT showed the lowest WHC (*p* < 0.05), indicating that the artificial muscles had better water retention (Fig. 8B). Among the artificial muscles, the SPI sample had a significantly lower WHC at 98.30%, whereas no significant differences were observed among samples containing raw RPI and ultrasound-assisted PRI-Xyl conjugates. CONT exhibited the significantly highest shrinkage rate of 58.02% during cooking (Fig. 8C). At high temperatures, muscle shrinkage in meat reduces WHC and increases cooking loss [41]. The shrinkage rates of the artificial muscles ranged from 4.57% to 7.79%, indicating greater dimensional stability than CONT.

### 3.7 Textural properties

**Fig. 9:**
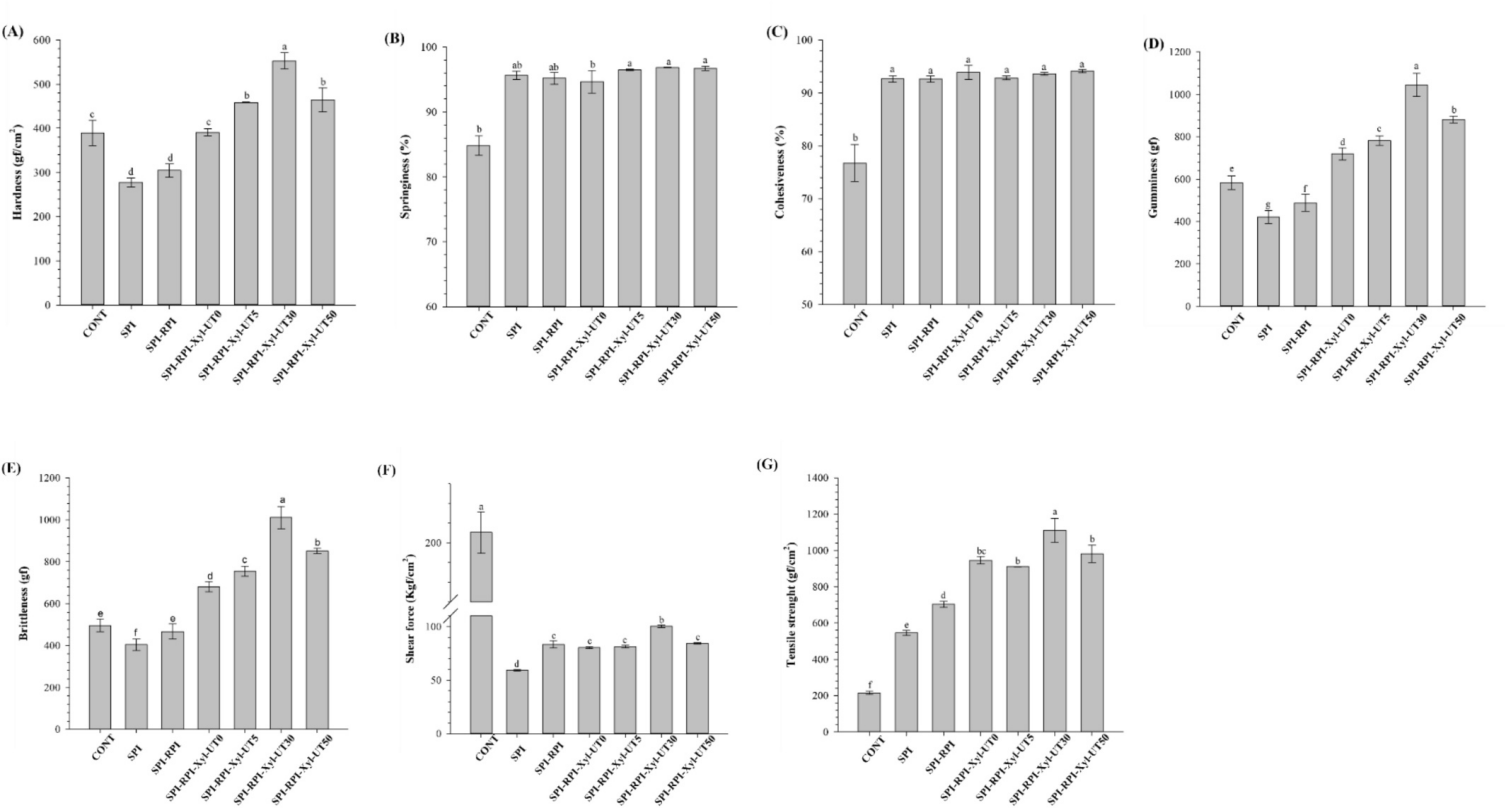
Hardness (A), springiness (B), cohesiveness (C), gumminess (D), brittleness (E), shear force (F), and tensile strength (G) of beef (CONT), and artificial muscles with varying contents of soy protein isolate (SPI), rice protein isolate (RPI), and d-xylose (Xyl), and different ultrasonic times (UT). Values are mean ± SD (n = 3). Different letters indicate significant differences (*p* < 0.05).

Textural properties of CONT, and artificial muscles containing different contents of SPI, RPI, and ultrasound-assisted RPI-Xyl conjugates are presented in Fig. 9. Optimizing the texture of meat analogs is essential for reproducing the sensory characteristics of CONT [30]. SPI and SPI-RPI samples showed lower hardness than CONT, whereas samples treated with ultrasound-assisted glycosylation exhibited significantly higher hardness (Fig. 9A). Wen et al. [46] reported similar results, demonstrating increased mechanical strength in meat analogs with glycosylation. The hardness values increased with UT and then decreased at UT 50 min. Peng et al. [69] found that meat analogs with increased disulfide bond formation exhibited greater firmness because these bonds maintained protein structure and enhanced protein cross-linking. Consequently, SPI-RPI-Xyl-UT30 with higher S‒S content achieved the highest hardness value after ultrasound treatment (*p* < 0.05). Gumminess and brittleness values exhibited trends similar to hardness (Fig. 9D and E). Gumminess represents the force required to disintegrate semi-solid food for swallowing and is strongly influenced by hardness. Brittle samples generally exhibit low cohesiveness but high fracturability [70]. Springiness and cohesiveness of the artificial muscles were significantly higher than those of CONT (Fig. 9B and C). Artificial muscles containing RPI-Xyl conjugates showed increased brittleness and internal strength, indicating a firmer and chewier texture. Appropriate ultrasonic treatment promoted intermolecular cross-linking and enhanced water retention (Fig. 8A), thereby improving textural properties. However, the shear force of CONT was significantly higher than that of the artificial muscles (Fig. 9F). The shear deformation behavior of meat is mainly determined by myofibrillar proteins and connective tissue [71]. Unlike meat, the artificial muscles had porous and heterogeneous microstructures (Fig. 6), resulting in low shear force values. Kim et al. [39] also reported that lower shear force observed in seafood analogs was attributed to differences in proteins composition. Tensile strength was significantly higher in glycosylated samples, particularly in SPI-RPI-Xyl-UT30 (Fig. 9G). During the glycosylation reaction, Xyl can form a stronger and more stable cross-linking network because of its high reactivity, thereby enhancing structural strength [29]. Overall, ultrasound-assisted glycosylation effectively modulated the textural properties of 3D-printed artificial muscles for meat analog applications.

## 4 Conclusion

In this study, ultrasound-assisted glycosylation was applied to modify RPI-Xyl conjugates and develop 3D-printed artificial muscles using SPI-based protein inks. RPI-Xyl conjugates prepared under different ultrasonic treatment times were incorporated into protein-based inks, and printing parameters were optimized using RSM based on their rheological properties. Among the rheological parameters, viscosity and yield stress were closely associated with extrusion behavior and shape retention, supporting flow rate optimization for each ink. Ultrasonic treatment promoted glycosylation and the Maillard reaction between RPI and Xyl, contributing to the heat-induced color transition of artificial muscles from red to brown. In addition, ultrasound-assisted glycosylation modified the secondary and tertiary structures of proteins in the artificial muscles. In particular, UT30 enhanced protein solubility and disulfide bond formation, whereas excessive ultrasonic treatment at UT50 reduced protein functionality, likely due to partial denaturation and aggregation. These structural changes were reflected in the cooked quality of the artificial muscles. SPI-RPI-Xyl-UT30 exhibited improved water retention, hardness, cohesiveness, elasticity, and tensile strength compared with CONT. Overall, this study demonstrates that ultrasound-assisted glycosylation can regulate the structure–function properties of protein-based inks for 3D-printed meat analogs. Further studies are needed to improve flavor-related attributes, particularly juiciness, through the incorporation of artificial fats and strategies to reduce fat release during cooking.

## CRediT authorship contribution statement

**Jung Soo Kim:** Writing – review & editing, Writing – original draft, Visualization, Project administration, Methodology, Investigation, Funding acquisition, Formal analysis, Conceptualization. **Jiyoon Kim:** Writing – review & editing, Visualization, Validation, Methodology, Investigation, Formal analysis, Data curation. **Kwang-Deog Moon:** Supervision, Resources, Funding acquisition.

## Declaration of competing interest

The authors declare that they have no known competing financial interests or personal relationships that could have appeared to influence the work reported in this paper.

## Appendix A Supplementary data

Supplementary Data 1 Supplementary figures (preliminary experiments on ultrasound-assisted glycosylation reactions, SDS-PAGE profiles, and FT–IR spectrum) and a supplementary table (experimental data based on face-centered central composite design).
