# Supplementary material for: Development of 3D-printed artificial muscles for meat analogs: structural and functional changes modulated by ultrasound-assisted glycosylation of rice protein with d-xylose
Source: Ultrason Sonochem. 2026 Aug 1;132:107947. doi: 10.1016/j.ultsonch.2026.107947 (PMC13487397; doi:10.1016/j.ultsonch.2026.107947)
Supplement: Supplementary file 1 [file mmc1.docx]

**Development of 3D-printed artificial muscles for meat analogs: Structural and functional changes modulated by ultrasound-assisted glycosylation of rice protein with d-xylose**

**Fig. S1. Protein solubility (A), sulfhydryl group and disulfide bond (B), and total soluble solids (C) on the ultrasound-assisted glycosylation as a function of RPI-to-Xyl mass ratio. Ultrasonic power and time were set to 100 W and 5 min, respectively. Values are mean±SD (n=3). Different letters indicate significant differences (*p*<0.05).**

**Fig. S2. Protein solubility (A), sulfhydryl group and disulfide bond (B), and total soluble solids (C) on the ultrasound-assisted glycosylation according to ultrasonic powder. RPI-to-Xyl mass ratio was 1:2, and ultrasonic time was 5 min. Values are mean±SD (n=3). Different letters indicate significant differences (*p*<0.05).**

**Fig. S3. Protein solubility (A), sulfhydryl group and disulfide bond (B), and total soluble solids (C) on the ultrasound-assisted glycosylation according to ultrasonic time. RPI-to-Xyl mass ratio was 1:2, and ultrasonic power was 500 W. Values are mean±SD (n=3). Different letters indicate significant differences (*p*<0.05).**

**Fig. S4. SDS-PAGE profiles (A) of rice protein isolate (RPI) and glycosylated products of RPI and d-xylose (Xyl) with different ultrasonic times (UT). M: standard protein marker; line 1: raw RPI; line 2: PRI-Xyl-UT0; line 3: RPI-Xyl-UT5; line 4: RPI-Xyl-UT30; line 5: RPI-Xyl-UT50. FT–IR spectrum (B) of raw soy protein isolate (SPI), raw RPI, and pure Xyl.**

**Table S1. Experimental data based on face-centered central composite design (FCCD) model for printed linewidth as a function of flow rate (%) and printing speed (mm/sec).**

| **Run order** | **Independent variables** | | | **Samples and response (printed linewidth, mm)** | | | | | |
| --- | --- | --- | --- | --- | --- | --- | --- | --- | --- |
|  | **Flow rate (%)** | **Printing speed (mm/sec)** | **SPI** | | **SPI-RPI** | **SPI-RPI-Xyl-UT0** | **SPI-RPI-Xyl-UT5** | **SPI-RPI-Xyl-UT30** | **SPI-RPI-Xyl-UT50** |
| **1** | -1 (25) | -1 (5) | 2.54±0.01 | | 2.43±0.01 | 2.51±0.01 | 2.49±0.01 | 2.47±0.02 | 2.55±0.01 |
| **2** | 1 (35) | -1 (5) | 3.44±0.02 | | 3.06±0.02 | 3.25±0.01 | 3.21±0.01 | 3.16±0.01 | 3.24±0.01 |
| **3** | -1 (25) | 1 (15) | 2.34±0.01 | | 2.22±0.02 | 2.29±0.01 | 2.28±0.01 | 2.26±0.01 | 2.28±0.01 |
| **4** | 1 (35) | 1 (15) | 2.85±0.01 | | 2.70±0.01 | 2.81±0.02 | 2.79±0.01 | 2.77±0.01 | 2.84±0.01 |
| **5** | -1 (25) | 0 (10) | 2.44±0.01 | | 2.33±0.01 | 2.40±0.01 | 2.38±0.01 | 2.36±0.01 | 2.43±0.02 |
| **6** | 1 (35) | 0 (10) | 3.24±0.01 | | 2.84±0.01 | 2.96±0.01 | 2.94±0.01 | 2.90±0.02 | 3.02±0.01 |
| **7** | 0 (30) | -1 (5) | 2.96±0.01 | | 2.74±0.01 | 2.91±0.01 | 2.86±0.01 | 2.85±0.01 | 2.92±0.01 |
| **8** | 0 (30) | 1 (15) | 2.62±0.01 | | 2.51±0.02 | 2.57±0.01 | 2.56±0.01 | 2.54±0.01 | 2.59±0.01 |
| **9-13** | 0 (30) | 0 (10) | 2.76±0.02 | | 2.64±0.01 | 2.74±0.00 | 2.73±0.00 | 2.68±0.01 | 2.76±0.01 |

Samples are protein-based inks with varying contents of soy protein isolate (SPI), rice protein isolate (SPI), and d-xylose (Xyl), and with different ultrasonic times (UT). Other parameters, including nozzle tip diameter (2.3 mm), layer height (1.5 mm), and printing temperature (25℃), were fixed. Values are mean±SD (n=3).
